# Treatment with Ascorbyl Glucoside–Arginine Complex Ameliorates Solar Lentigos

**DOI:** 10.3390/ijms252413453

**Published:** 2024-12-15

**Authors:** Mariko Takada, Kayoko Numano, Masahiko Nakano, Akio Yamamoto, Genji Imokawa

**Affiliations:** 1 Center for Bioscience Research and Education, Utsunomiya University, Tochigi 321-8505, Japan; mrktkd0711@cc.utsunomiya-u.ac.jp; 2 Ebisu Ray Clinic, Tokyo 150-0013, Japan; kayoko.n21@icloud.com; 3 Cosmetic Research Center, Doctor’s Choice Co., Ltd., Tokyo 102-0071, Japan; mnakano@drs-choice.co.jp (M.N.); yamamoto@drs-choice.co.jp (A.Y.)

**Keywords:** ascorbyl glucoside–arginine complex, solar lentigos, double-blind half-face study, pigmentation, L value, melanin index

## Abstract

Little is known about the anti-pigmenting effects of skin-whitening agents on solar lentigos (SLs). To characterize the anti-pigmenting effects of a newly designed derivative ascorbyl glucoside–arginine complex (AGAC) on SLs, lotions with or without 28% AGAC were applied twice daily for 24 weeks in a double-blind half-face study of 27 Japanese females with SLs. The pigmentation scores and skin colors of previously selected SLs on the right and left sides of the faces of the subjects were evaluated using a photo-scale, a color difference meter and a Mexameter. Treatment with the test lotion elicited a significant decrease in pigmentation scores at 24 weeks compared to week 0, with a significant decrease in pigmentation scores at 24 weeks compared to the placebo lotion. In the test lotion-treated SLs, the lightness (L) and melanin index (MI) values that reflect the pigmentation level significantly increased and decreased, respectively, at 12 and 24 weeks of treatment compared to week 0. Comparisons of increased L values or decreased MI values between the test and placebo lotion-treated SLs demonstrated that the test lotion-treated SLs had significantly higher increased L or decreased MI values than the placebo lotion-treated SLs both at 12 and 24 weeks of treatment. The sum of our results strongly indicates that AGAC is distinctly effective in ameliorating the hyperpigmentation levels of SLs at a level visibly recognizable by the subjects, without any hypo-pigmenting effects or skin problems.

## 1. Introduction

Solar lentigos (SLs) are hyperpigmented lesions that frequently occur on sun-exposed skin, especially on the face and the dorsum of the hands of Asian subjects [1]. Based on the frequency of the final diagnosis of patients with various pigmentary disorders in Japan, SLs have the highest incidence, occurring in approximately 60% of all patients with hyperpigmentary disorders, while melasma and post-inflammatory hyperpigmentation (including ultraviolet B (UVB) melanosis) occur in as few as 5.2% and 3.3% of patients, respectively [1]. 

In general, hyperpigmentary disorders, including UVB melanosis, SLs and melasma, are targeted by anti-pigmenting agents. However, establishing an effective anti-pigmenting treatment for SLs is challenging, especially for dark-skinned individuals, because the treatment needs to reduce the hyperpigmentation without causing any undesirable hypopigmentation or contact irritation in the surrounding normal pigmented skin. Hydroquinone (HQ) is one of the most effective drugs to treat hyperpigmentary disorders, including SLs [2,3,4,5,6], but treatment with HQ often causes skin irritation [7,8,9,10,11,12,13]. Therefore, there have been no available anti-pigmenting agents capable of ameliorating SLs without causing any skin problems even during long-term treatments.

Although many skin-whitening agents are available [14,15,16,17,18,19,20,21,22,23,24,25,26,27,28,29,30,31], some of which are targeted and approved especially to treat UVB-induced hyperpigmentation but not SLs in Japan, little is known about the potential anti-pigmenting effects of those skin-whitening agents on SLs because clinical evaluations of SLs are not required for approval as a skin-whitening agent in Japan. Excluding kojic acid and rhododendrol, which have been reported to elicit hypopigmentation on the faces of dark-skinned individuals [32,33,34,35], other skin-whitening agents approved in Japan are suitable candidates for investigating their potential anti-pigmenting effects on SLs because they have been proven to be very safe in terms of hypopigmentation and skin irritation since they have been commercially available for a long period of time. Among those whitening agents, ascorbic acid derivatives are thought to be invaluable agents, especially from a skin safety point of view. 

L-ascorbate-2-phosphate Mg (AMP), a skin-whitening agent first approved as a cosmetic medicine in Japan, was reported to have a significant ameliorating effect on chloasma by acting as a tyrosinase inhibitor after it is enzymatically degraded by intrinsic epidermal phosphatases to release free ascorbic acid [36,37,38,39,40,41,42,43]. L-ascorbate-2-phosphate trisodium salt (APS) is another approved whitening agent in Japan [36,44,45]. APS is a modified derivative of AMP that improves its stability, namely its aggregation due to the Mg salt [46]. Ascorbyl glucoside (AG) is also a whitening agent approved in Japan that has been documented to have a depigmenting effect on UVB hyperpigmentation by acting as a tyrosinase inhibitor itself or after it is converted by intrinsic epidermal glucosidase to free ascorbic acid [31]. Based on the requirement for approval of skin-whitening agents in Japan, it is well established that the topical application of AMP, APS or AG for 21 days on UVB (2MED)-exposed human skin significantly inhibits UVB-increased pigmentation, measured as L values at 21 days post-UVB irradiation. However, there were no published data on the anti-pigmenting effect of topical treatment with APS, AMP or AG on SLs in a double-blind half-face study, although a whole-face study using AMP on SLs for 3 months was reported to have some efficacy [37], but that study was flawed due to the lack of a placebo control. We recently reported, for the first time, that in a double-blind half-face study of 27 Japanese female subjects with SLs who used lotions with or without 6% APS (test lotion and placebo lotion, respectively) applied twice a day for 24 weeks, APS had a weak but significant anti-pigmenting effect on SLs and also a significant whitening effect even on normally pigmented non-lesional surrounding skin (NLS) [47]. However, that clinical study was not satisfactory for clinical evaluation because there was no significant difference in the pigmentation scores of SLs judged by a dermatologist between the test and placebo lotions. Because of established information on skin safety for AG [48,49], this prompted us to modify AG by making a complex with arginine to reduce its acidity and to increase the concentration used to 28%, which is the highest limit due to the lotion properties. A benefit of using an ascorbyl glucoside–arginine complex (AGAC) at 28% is its neutral pH of 6.5, compared to pH 1.98 for ascorbyl glucoside at 5% and pH 2.2~2.5 for ascorbic acid at 5%, because such acidity generally elicits a stinging sensation on faces, which frequently leads to an itchy sensation and skin irritation partly due to their chemical instability. Further, safety assessments of AGAC, including the Ames test, 24 h closed patch test, eye mucosal irritation test and cytotoxicity test, have proven its general safety as a topical compound [50]. This evidence prompted us to use AGAC at 28% as a therapeutic treatment on SLs for a long period of time. 

In this study, we conducted a double-blind half-face study of 27 Japanese female subjects with SLs who used lotions with or without 28% AGAC (test lotion and placebo lotion, respectively) applied twice a day for 24 weeks. The results of this study show that repeated topical treatment with AGAC has a significant anti-pigmenting effect on SLs, with a significant difference in the pigmentation scores of SLs judged by a dermatologist between the test and placebo lotions. These results were corroborated by objective instrumental evaluations using a color difference meter and a Mexameter, and show that there was a significant whitening effect even on normally pigmented NLS without any hypo-pigmenting effects at the skin color level.

## 2. Results

### 2.1. Visible Pigmentation Level of SLs

**Figure 1:**
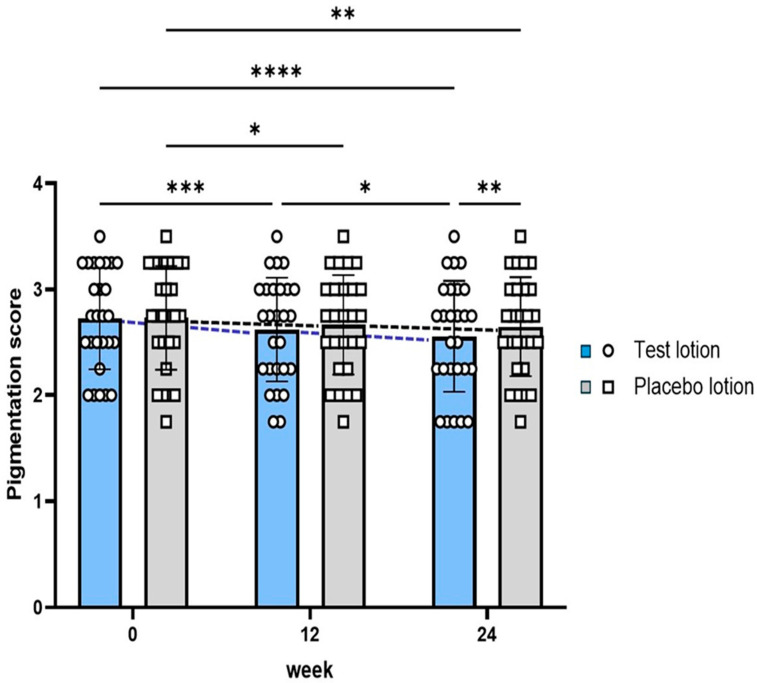
Evaluation of pigmentation scores of one previously selected SL at 0, 12 and 24 weeks of treatment. Bars represent means ± SD. n = 27; ****: *p* < 0.0001; ***: *p* < 0.001; **: *p* < 0.01; and *: *p* < 0.05 according to ANOVA followed by Tukey’s comparison test.

Topical applications of lotions with or without 28% AGAC (test lotion and placebo lotion, respectively) were carried out twice daily for 24 weeks on the entire right and left sides of the face of each subject. In the clinical and instrumental evaluations of changes in the pigmentation levels of SLs, we found that the comparative paired measurements between halves of faces focusing on one SL with a similar pigmentation level were more appropriate, especially for paired comparisons, than focusing on many SLs with different pigmentation levels [47]. Therefore, in this study, the pigmentation levels of a previously selected SL on the right and left sides of each subject’s face were assessed by a dermatologist at 0, 12 and 24 weeks using a photo-scale ranging from 1.0 to 5.0 (KN), and by using a color difference meter and a Mexameter MX18. The results of the clinical evaluation (Figure 1) indicate that although the placebo lotion-treated SLs had significant (*p* < 0.05) decreases in pigmentation scores at 12 weeks compared to week 0, the test lotion-treated SLs had more marked significant (*p* < 0.001 and *p* < 0.0001) decreases in pigmentation scores at 12 and 24 weeks compared to week 0, accompanied by a significant (*p* < 0.01) decrease in pigmentation scores at 24 weeks compared to the placebo lotion-treated SLs. These results suggest that there was a weak but distinct anti-pigmenting effect of the test lotion on SLs at a visible clinical level. 

### 2.2. Clinical Photographs of a Solar Lentigo (SL) and Non-Lesional Surrounding Skin (NLS)

**Figure 2:**
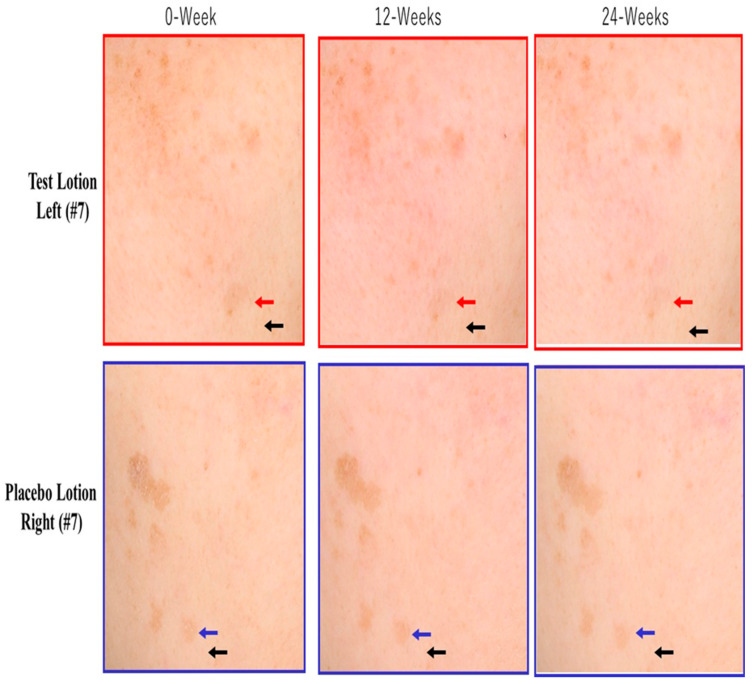
Clinical photographs of a SL and one non-lesional surrounding skin (NLS) area. Red arrows indicate a test lotion-treated SL on the left side of the face of subject #7. Blue arrows indicate a placebo lotion-treated SL on the right side of the face of subject #7. Black arrows indicate one test or placebo lotion-treated non-lesional surrounding skin (NLS) area on the right or left side of the face of subject #7.

Representative photographs of the face of subject #7 (Figure 2) at 0, 12 and 24 weeks of treatment showed that the pigmentation level of a test lotion-treated SL (indicated by red arrows) slightly decreased at 24 weeks compared to week 0. In contrast, the pigmentation level of a placebo lotion-treated SL (indicated by blue arrows) at 24 weeks remained unchanged compared to week 0.

### 2.3. L and MI Values of SLs

**Figure 3:**
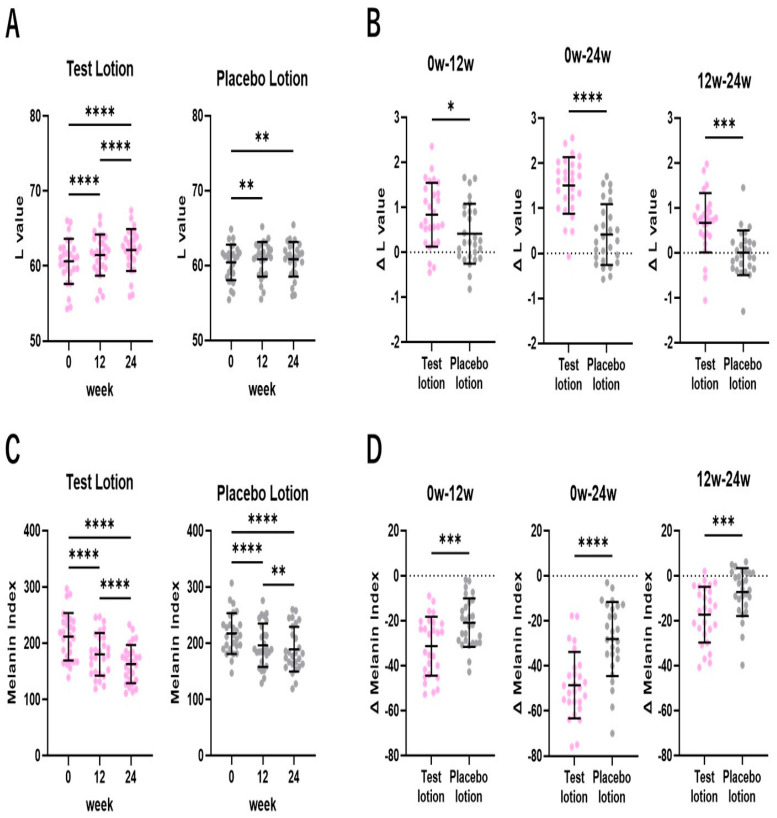
Changes in L values and MI values of SLs after treatment for 24 weeks. (**A**,**C**): time course study. Bars represent means ± SD. n = 27. ****: *p* < 0.0001; and **: *p* < 0.01 according to ANOVA followed by Tukey’s comparison test. (**B**,**D**): increased (Δ) L values and decreased (Δ) MI values between 0 and 12, 0 and 24, and 12 and 24 weeks. Bars represent means ± SD. n = 27. ****: *p* < 0.0001; ***: *p* < 0.001; and *: *p* < 0.05 according to paired *t* test.

To evaluate anti-pigmenting effects on SLs, lightness (L) and melanin index (MI) values that reflect pigmentation levels [51,52,53,54,55] were measured in the test lotion- or the placebo lotion-treated SLs using a color difference meter and a Mexameter MX18, respectively, at 0, 12 and 24 weeks of treatment. In these objective instrumental evaluations, an increase in L values and a decrease in MI values reflect a decrease in pigmentation level [47,51,52,53,54]. In the test lotion-treated SLs, the L values significantly (*p* < 0.0001) increased at 12 and 24 weeks of treatment compared to week 0, with a significant (*p* < 0.0001) increase between 12 and 24 weeks (Figure 3A). In the placebo lotion-treated SLs, the L values also significantly (*p* < 0.01) increased at 12 and 24 weeks of treatment compared to week 0, but without any significant increase between 12 and 24 weeks (Figure 3A). Comparisons of the increased (Δ) L values between the test and placebo lotion-treated SLs demonstrated that the test lotion-treated SLs had significantly (*p* < 0.05, *p* < 0.0001 and *p* < 0.001) higher ΔL values than the placebo lotion-treated SLs at 0~12, 0~24 and 12~24 weeks, respectively, of treatment (Figure 3B). While values over 2.0 ΔL and 1.6 ΔL are, respectively, levels distinctly and slightly recognizable by subjects [47], the ratio of subjects with values over 2.0 ΔL or 1.6 ΔL for SLs was 6 or 15 in 27 for the test lotion and 0 or 1, respectively, in 27 for the placebo lotion at 0~24 weeks (Figure 3B).

The MI values were significantly (*p* < 0.0001) decreased at 12 and 24 weeks of treatment compared to week 0 in the test lotion-treated SLs, with a significant (*p* < 0.0001) decrease between 12 and 24 weeks (Figure 3C), while in the placebo lotion-treated SLs, the MI values also significantly (*p* < 0.0001) decreased at 12 and 24 weeks of treatment compared to week 0, with a significant (*p* < 0.01) decrease between 12 and 24 weeks (Figure 3C). Comparisons of the decreased (Δ) MI values between the test and placebo lotion-treated SLs demonstrated that the test lotion-treated SLs had significantly (*p* < 0.001, *p* < 0.0001 and *p* < 0.001) lower ΔMI values than the placebo lotion-treated SLs at 0~12, 0~24 and 12~24 weeks, respectively, of treatment (Figure 3D). While a value over 50 ΔMI is a level distinctly recognizable by subjects (47), the ratio of subjects with a value over 50 ΔMI for SLs was 13 in 27 for the test lotion and 2 in 27 for the placebo lotion at 0~24 weeks (Figure 3D). These findings suggest that the test lotion, but not the placebo lotion, has a weak but distinct anti-pigmenting effect on SLs at both the color difference and the MI levels.

### 2.4. L and MI Values of One NLS Area

**Figure 4:**
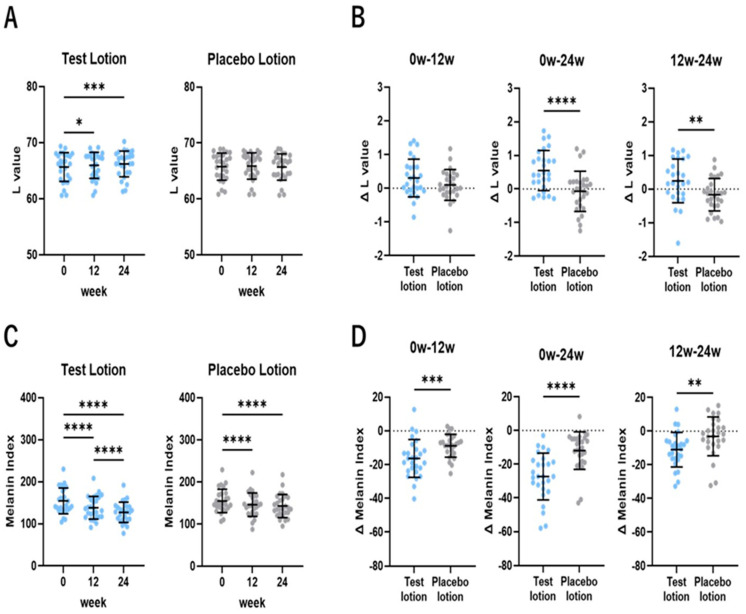
Changes in L values and MI values of NLS after treatment for 24 weeks. (**A**,**C**): time course study. Bars represent means ± SD. n = 27, ****: *p* < 0.0001; ***: *p* < 0.001; **: *p* < 0.01; and *: *p* < 0.05 according to Tukey’s comparison test, followed by ANOVA. (**B**,**D**): increased (Δ) L values and decreased (Δ) MI values between 0 and 12, 0 and 24, and 12 and 24 weeks. Bars represent means ± SD. n = 27; ****: *p* < 0.0001; and ***: *p* < 0.001 according to paired *t* test.

To evaluate the whitening effects on one NLS area, L and MI values were measured in the test lotion- or the placebo lotion-treated NLS area using a color difference meter and a Mexameter MX18, respectively, at 0, 12 and 24 weeks of treatment. In test lotion-treated NLS, L values significantly (*p* < 0.05 and *p* < 0.001) increased at 12 and 24 weeks, respectively, of treatment compared to week 0 (Figure 4A), whereas in the placebo lotion-treated NLS, L values did not increase at 12 and 24 weeks of treatment compared to week 0. Comparisons of ΔL values between test and placebo lotion-treated NLS demonstrated that test lotion-treated NLS had a significantly (*p* < 0.0001 and *p* < 0.01) higher ΔL value than placebo lotion-treated NLS at 0~24 and 12~24 weeks of treatment (Figure 4B). While a value over 1.6 ΔL is a level slightly recognizable by subjects [47], the ratio of subjects with a value over 1.6 ΔL for NLS was 3 in 27 for the test lotion and 0 in 27 for the placebo lotion at 0~24 weeks. In test lotion-treated NLS, MI values significantly (*p* < 0.0001) decreased at both 12 and 24 weeks of treatment compared to week 0, with a significant (*p* < 0.0001) decrease between 12 and 24 weeks (Figure 4C), while in placebo lotion-treated NLS, MI values also significantly (*p* < 0.0001) decreased at both 12 and 24 weeks of treatment compared to week 0, but that was not accompanied by any significant decrease between 12 and 24 weeks. Comparisons of ΔMI values between test and placebo lotion-treated NLS demonstrated that test lotion-treated NLS had a significantly (*p* < 0.001, *p* < 0.0001 and *p* < 0.01) lower ΔMI value than placebo lotion-treated NLS at 0~12, 0~24 and 12~24 weeks, respectively, of treatment (Figure 4D). While a value over 30 ΔMI is a level slightly recognizable by subjects [47], the ratio of subjects with a value over 30 ΔMI for NLS was 10 in 27 for the test lotion and 2 in 27 for the placebo lotion at 0~24 weeks. These findings suggest that the test lotion, but not the placebo lotion, has a weak but distinct whitening effect on NLS areas at the color difference and MI levels.

### 2.5. Correlations Between L and MI Values in SLs and NLS

**Figure 5:**
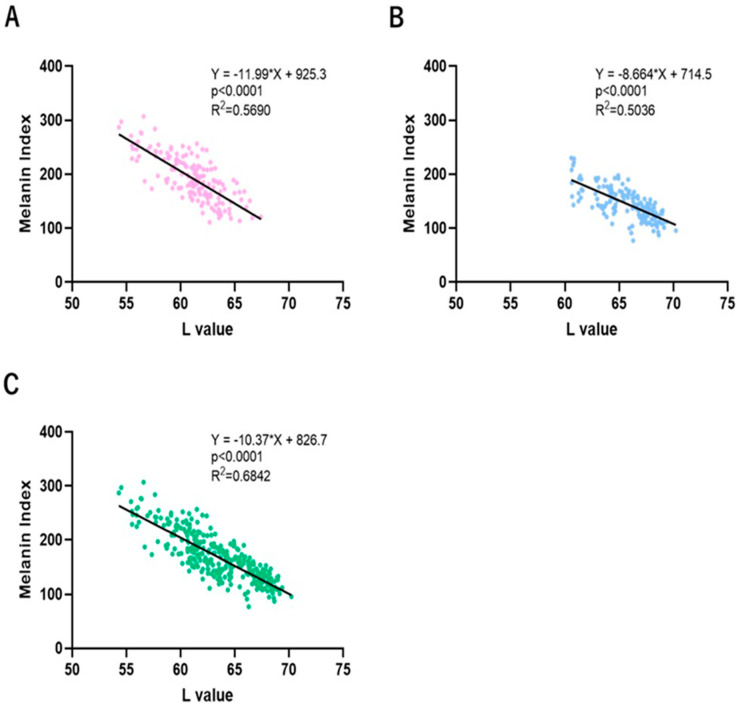
Correlations between L and MI values in SLs, NLS and SLs + NLS during this clinical study. (**A**): Correlation between L and MI values in SLs. (**B**): Correlation between L and MI values in NSL areas. (**C**): Correlation between L and MI values in SLs + NSL.

**Figure 6:**
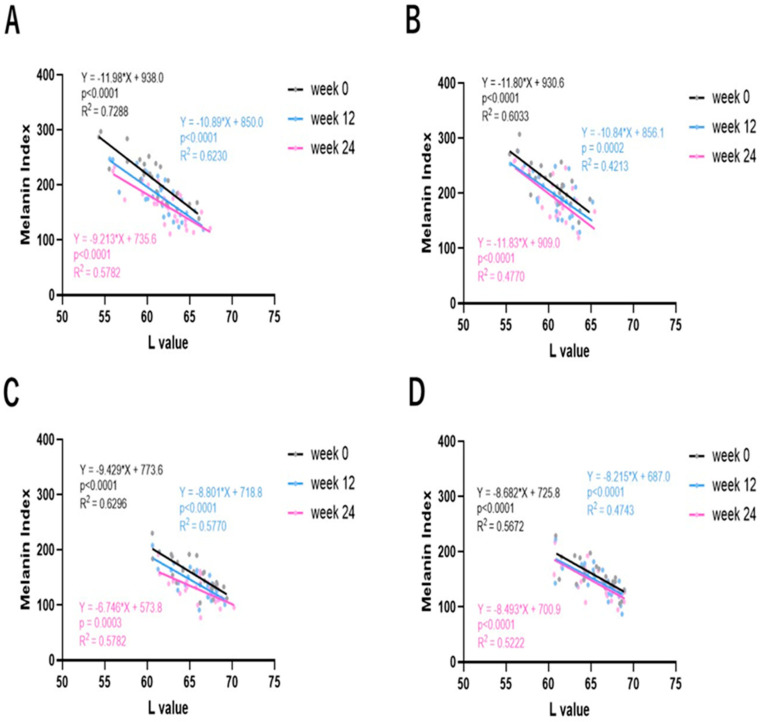
Correlations between L and MI values in SLs and NLS at 0, 12 and 24 weeks of treatment with test or placebo lotion. (**A**): Correlation between L and MI values in test lotion-treated SLs. (**B**): Correlation between L and MI values in placebo lotion-treated SLs. (**C**): Correlation between L and MI values in test lotion-treated NSL. (**D**): Correlation between L and MI values in placebo lotion-treated NSL.

Correlation plots between L and MI values for SLs, NLS and SLs + NLS indicated that L and MI values have good correlations in SLs, NLS and SLs + NLS (Figure 5). Figure 6 shows correlation plots between L and MI values in the test and placebo lotion-treated SLs and NLS at 0, 12 and 24 weeks of treatment. In the test lotion-treated SLs (Figure 6A), while the slopes of the correlation plots are similar at 0, 12 and 24 weeks of treatment, their intercepts distinctly decrease, with a parallel shift throughout 0, 12 and 24 weeks of treatment. These results indicate that the test lotion has a distinct potential to increase L values and decrease MI values, reflecting its distinct anti-pigmenting effects. In contrast, treatment with the placebo lotion does not have a tendency similar to the test lotion-treated SLs (Figure 6B). In test lotion-treated NLS (Figure 6C), although the slopes of the correlation plots are similar at 0, 12 and 24 weeks of treatment, their intercepts distinctly decrease, with a parallel shift throughout 0, 12 and 24 weeks of treatment. These results indicate that the test lotion has a distinct potential to increase L values and decrease MI values, reflecting its whitening effects on NLS. In contrast, in placebo lotion-treated NLS, the correlation plots do not have such a tendency (Figure 6D).

### 2.6. General Clinical Evaluation

**Figure 7:**
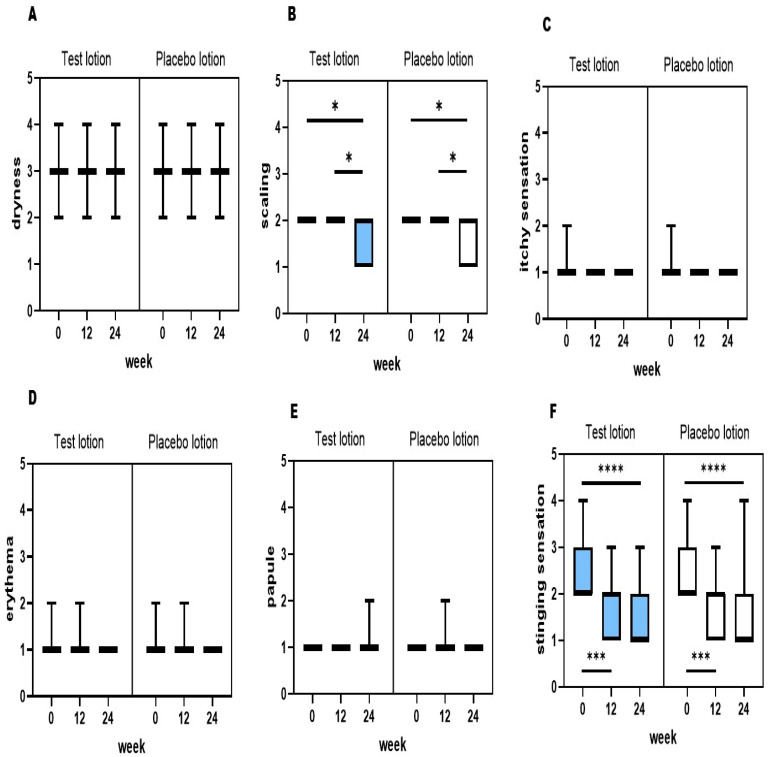
The clinical effects of the test and placebo lotions on the facial skin symptoms of subjects with SLs. (**A**): Dryness, (**B**): scaling, (**C**): itchy sensation, (**D**): erythema, (**E**): papules, (**F**): stinging sensation. Clinical scoring was performed at 0, 12 and 24 weeks according to the criteria described in the Materials and Methods section. n = 27; *: *p* < 0.05; ***: *p* < 0.001; and ****: *p* < 0.0001, compared to week 0. All data were analyzed using a Friedman test and Dunn’s multiple comparisons test.

During the test period of 24 weeks, no skin problems occurred, including skin irritation or hypopigmentation, in the test lotion- or the placebo lotion-treated skin. Therefore, the test lotion was considered by a trained dermatologist (KN) to be safe for long-term use. A general clinical evaluation was also performed on both the entire right and left sides of the face of each subject by a trained dermatologist (KN) at 0, 12 and 24 weeks of treatment. This evaluation demonstrated that treatments with the test lotion or the placebo lotion induced a significant decrease (*p* < 0.05) in the scores of scaling a at 24 weeks (Figure 7B), whereas the same treatments did not cause any changes in dryness, itchiness, erythema or papules at 12 and 24 weeks (Figure 7A,C–E). Stinging sensations were significantly (*p* < 0.001) ameliorated after treatment with the test lotion and the placebo lotion for 12 and 24 weeks, compared with week 0 (Figure 7F). These findings indicate that both the test and placebo lotions have a distinct ameliorating effect on scaling and stinging sensation without any skin irritation.

### 2.7. Clinical Evaluation of Skin Redness Based on Erythema Index Values

**Figure 8:**
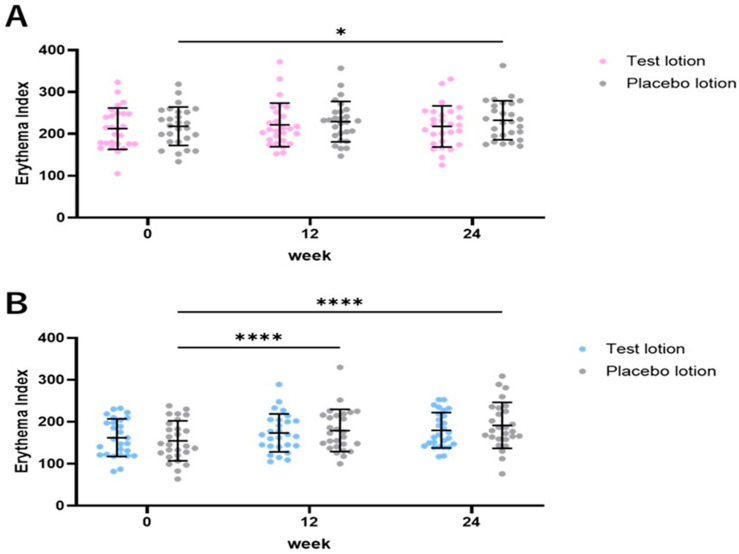

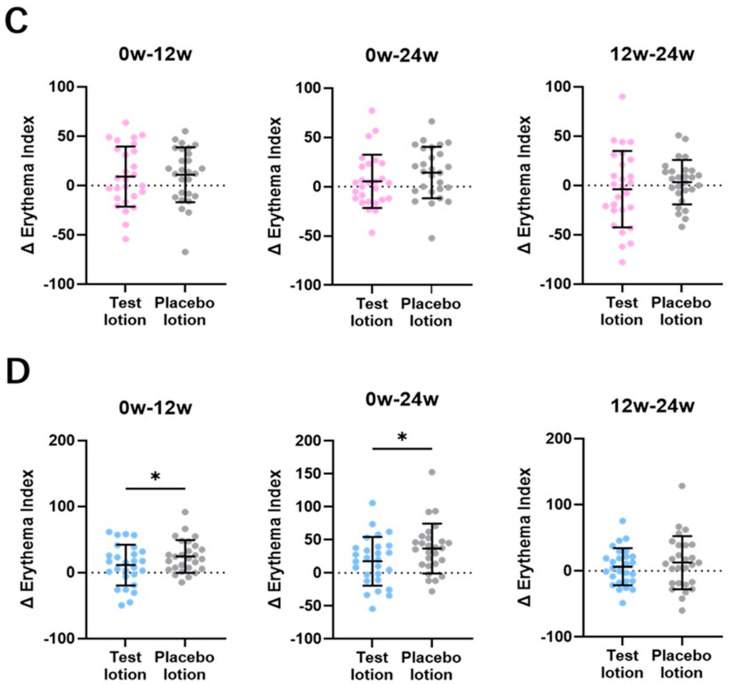
The clinical effects of the test and placebo lotions on the intensity of skin redness, measured as the erythema index with a Mexameter MX18. (**A**): Time course study in the lotion-treated SLs. Bars represent means ± SD. n = 27 and *: *p* < 0.05, according to ANOVA followed by Tukey’s comparison test. (**B**): Time course study in lotion-treated NLS. Bars represent means ± SD. n = 27 and ****: *p* < 0.0001, according to ANOVA followed by Tukey’s comparison test. (**C**): Increased (Δ) erythema index values between 0 and 12, 0 and 24, and 12 and 24 weeks in the lotion-treated SLs. Bars represent means ± SD. n = 27. (**D**): Increased (Δ) erythema index values between 0 and 12, 0 and 24, and 12 and 24 weeks in lotion-treated NSL. Bars represent means ± SD. n = 27 and *: *p* < 0.05 according to paired *t* test.

Since erythema index values obtained using a Mexameter MX18 can serve as a measure of skin redness [51], the erythema index values of the test lotion-treated SLs were measured at 0, 12 and 24 weeks of treatment and were compared with those of placebo lotion-treated SLs. In the test lotion-treated SLs, the erythema index values did not change at 12 and 24 weeks of treatment compared to week 0 (Figure 8A). In contrast, in the placebo lotion-treated SLs, the erythema index values significantly (*p* < 0.01) increased at 24 weeks of treatment compared to week 0. Comparisons of the increased (Δ) erythema index values between the test and placebo lotion-treated SLs demonstrated that the test lotion-treated SLs had similar Δ erythema index values compared to the placebo lotion-treated SLs at both 12 and 24 weeks of treatment (Figure 8C). In the test lotion-treated NLS areas, the erythema index values did not change at 12 and 24 weeks of treatment compared to week 0 (Figure 8B). However, in theplacebo lotion-treated NLS, the erythema index values significantly (*p* < 0.0001) increased at 12 and 24 weeks of treatment compared to week 0. Comparisons of the increased erythema index values between test and placebo lotion-treated NLS demonstrated that test lotion-treated NLS had significantly (*p* < 0.05) lower Δ erythema index values than placebo lotion-treated NLS at both 12 and 24 weeks of treatment (Figure 8D). These results indicate that the test lotion, but not the placebo lotion, has a weak but distinct potential to ameliorate skin redness, which may reflect a weak anti-inflammatory effect of the test lotion.

## 3. Discussion

AGAC is thought to function as a tyrosinase inhibitor following its conversion to ascorbic acid via its deglycosylation by epidermal α-glucosidases after it penetrates into the epidermis. α-Glucosidases are known to exist in the epidermis as protein glycosylation processing enzymes that can break the glucose of asparagine-linked carbohydrate moieties bound to proteins to release glucose in the Golgi area of keratinocytes [56]. In order for the action of tyrosinase inhibitors to be effective, it was essential to know whether the hyperpigmentation of SLs is accompanied by accentuated expression of the key melanogenic enzyme tyrosinase in the lesional melanocytes. It has already been reported that the hyperpigmentation in SLs occurs in concert with the up-regulated mRNA levels of tyrosinase in increased numbers of tyrosinase-positive melanocytes in the SL lesional epidermis [1]. 

The present double-blind half-face study of subjects with SLs demonstrated that the test lotion-treated SLs had more marked significant decreases than the placebo in pigmentation scores at 12 (*p* < 0.001) and 24 (*p* < 0.0001) weeks compared to week 0, accompanied by a significant decrease in pigmentation scores at 24 weeks (*p* < 0.01) compared to the placebo (Figure 1). It should be noted that significant decreases (*p* < 0.05) in pigmentation scores in the placebo lotion-treated SLs at 12 weeks compared to week 0 probably occurred due to seasonal changes in skin color from September to March during this clinical study. These results suggest that AGAC has a weak but distinct potential to ameliorate the clinical hyperpigmentation level of SLs. This clinical anti-pigmenting effect was corroborated by objective instrumental measurements using a color difference meter and a Mexameter. In these instrumental evaluations, although both the test and placebo lotions significantly increased the L values or decreased the MI values at 12 and 24 weeks of treatment, comparisons of the increased L (ΔL) values and the decreased (Δ) MI values between the test and placebo lotion-treated SLs demonstrated that the test lotion-treated SLs had significantly higher ΔL and ΔMI values than the placebo lotion-treated SLs at 0~12, 12~24 and 0~24 weeks of treatment (Figure 3). Since the significant anti-pigmenting effects of the placebo lotion might reflect seasonal changes in skin color from September to March during this clinical study, the significant differences observed in both the ΔL and ΔMI values at 12 and 24 weeks compared to week 0 between the test and placebo lotions suggest that AGAC has a distinct anti-pigmenting effect on SLs at the color difference and MI levels. Further, we found that the ratio of subjects with distinctly recognizable levels of over 2.0 ΔL or a 50 ΔMI value for SLs was 6 or 13 in 27 for the test lotion, and 0 or 2, respectively, in 27 for the placebo lotion at 0~24 weeks (Figure 3). These results strongly suggest that AGAC is distinctly effective in diminishing the hyperpigmentation levels of SLs at a level visibly recognizable by the subjects themselves. In the time course of anti-pigmenting effects, measured as L values, especially by the color difference meter, significant effects of the test lotion on SLs occurred in a step-by-step manner at 12 and 24 weeks of treatment, with significantly increased changes even at 12~24 weeks. In contrast, these significant effects of the placebo lotion also occurred at 12 and 24 weeks of treatment, but were not accompanied by any significant changes at 12~24 weeks (Figure 3A). These time course trends of the anti-pigmenting effects of the test lotion could provide an insight intopredicting more distinct anti-pigmenting effects possibly byfurther prolonged treatments with the test lotion. 

Of considerable interest is the fact that in test lotion-treated NLS, both the L and MI values significantly increased or decreased at 12 and 24 weeks of treatment compared to week 0 (Figure 4A,C). In contrast, in placebo lotion-treated NLS, the L values did not increase at 12 and 24 weeks of treatment compared to week 0 (Figure 4A), while the MI values significantly decreased at 12 and 24 weeks of treatment (Figure 4C). Although the significant decrease in MI values in placebo lotion-treated NLS seemed to occur due to the seasonal variation from September to March during this study, comparisons of increased L (ΔL) values or decreased MI (ΔMI) values between test and placebo lotion-treated NLS demonstrated that test lotion-treated NLS had significantly higher ΔL and lower ΔMI values than placebo lotion-treated NLS at 0~24 and 12~24 weeks of treatment (Figure 4B,D). Although increased or decreased levels of L or MI values occur at lower levels than 2.0 ΔL or 50 ΔMI (levels distinctly recognizable by the subjects) at 0~24 weeks, the ratio of subjects with a value over 1.6 ΔL or 30 ΔMI (levels slightly recognizable by the subjects) for NLS at 0~24 weeks was 3 and 10 in 27 for the test lotion, and 0 and 2 in 27 for the placebo lotion (Figure 4B,D). The sum of these findings indicates that the test lotion has a significantly higher whitening effect on NLS than the placebo lotion and suggests that AGAC has a weak but significant whitening effect on NLS at visibly recognizable levels.

It was of considerable interest to compare the anti-pigmenting effects on SLs and the whitening effects on NLS between AGAC and ASP, because the latter has been reported to have both anti-pigmenting and whitening effects in subjects with SLs [47]. Although both compounds have similar anti-pigmenting and whitening effects on SLs and NLS, respectively, as revealed by the evaluation of L and MI values, a major difference occurred at the clinical scoring level of pigmentation in SLs, in which AGAC but not ASP exhibited a significant (*p* < 0.0001) decrease in pigmentation scores at 24 weeks compared to week 0, accompanied by a significant (*p* < 0.01) decrease in pigmentation scores at 24 weeks compared to the placebo lotion-treated SLs (Figure 1). This indicates that AGAC is slightly superior to ASP from a clinical point of view, although the production cost is much higher for AGAC than for ASP. It is likely that the slight superiority of AGAC to ASP can be ascribed to the higher concentration used, i.e., 28% AGAC compared to 6% ASP, despite the fact that the rate of penetration into the epidermis is thought to be much higher for ASP than AGAC.

A major skin problem that can occur during the long-term topical application of anti-pigmenting agents is skin irritation, as is frequently observed for HQ [7,8,9]. Since such a long duration of topical applications is required to attain a distinct anti-pigmenting effect in dark-skinned individuals with SLs, the skin irritation that occurs during the treatment can be a major causative factor for not being able to continue the topical treatments. Therefore, general clinical evaluations of skin symptoms, including skin irritation, are important and were carried out in this study by a trained dermatologist (KN). These clinical evaluations demonstrated that, while the test lotion rather significantly ameliorated scaling and stinging sensations at 24 weeks, there was no appearance of erythema, papules or itchiness during the 24 weeks of treatment. Further, based on the evidence that erythema index values measured using a Mexameter can serve as a reflection of skin redness due to hemoglobin levels in the blood [51], our evaluations revealed that the test lotion, but not the placebo lotion, has a distinct potential to diminish skin redness. These findings strongly suggest that AGAC could act as an anti-pigmenting agent with a weak anti-inflammatory effect.

## 4. Conclusions and Future Perspectives

The sum of the results of this study indicates that AGAC has a weak but significant anti-pigmenting effect on SLs and a significant whitening effect even on normally pigmented NLS without the risk of eliciting hypopigmentation or skin irritation. This provides skin safety at a sufficient level to use via topical application for a long period of time, which is an essential requirement to achieve distinct anti-pigmenting effects on SLs because AGAC treatment does not target causative factors (keratinocyte DNA damage and the subsequent secretion of tumor necrosis factor [1]) for developing solar lentigo. Thus, if you stop treatments, the SL will return to its original appearance. It is anticipated that our evidence will facilitate the usage of AGAC as a therapeutic treatment for SLs for a long period of time.

## 5. Materials and Methods

### 5.1. Test Materials

**Figure 9:**
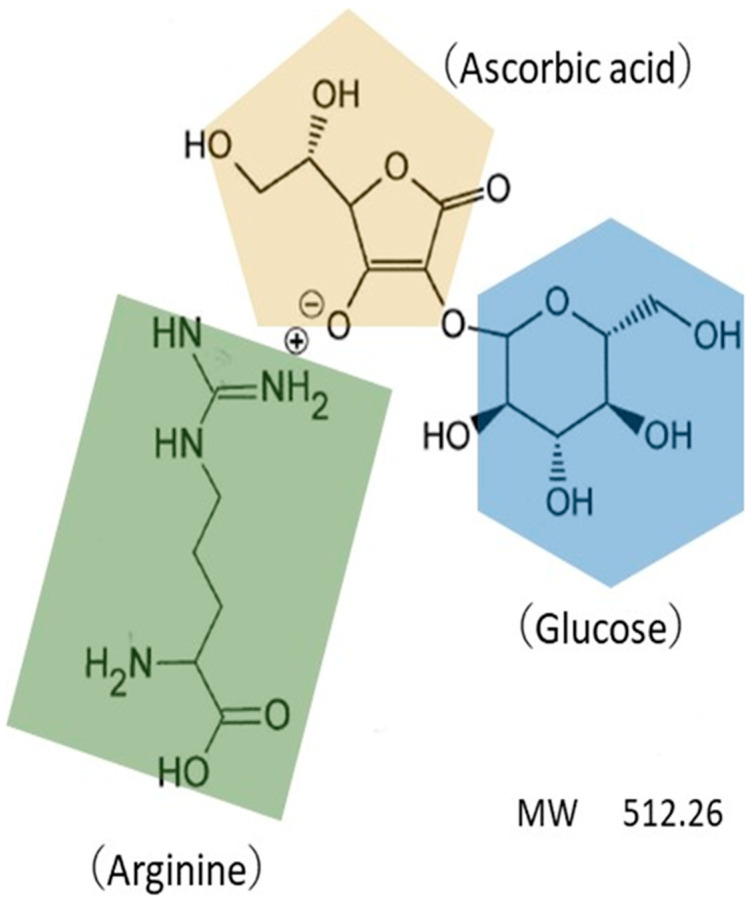
Chemical structure of AGAC.

The chemical structure of AGAC is shown in Figure 9. The test and placebo lotions were provided by Doctor’s Choice Co., Ltd. (Tokyo, Japan). The test lotion, a transparent thick liquid with pH 6.5, comprised 28% AGAC and other ingredients, as listed in Table 1. The placebo lotion comprised the same components as the test lotion except it did not include AGAC. Ascorbyl glucoside and arginine were purchased from Technoble Co., Ltd. (Osaka, Japan) and Technoscience Co., Ltd. (Kashiwa, Japan), respectively. AGAC has been reported to have general cutaneous safeness in a safety assessment [50].

### 5.2. Study Design

This study was performed from September 2023 to March 2024, at the Ebisu Skin Research Center, Inforward Co., LTD, Tokyo, Japan. Following screening by a dermatologist, 27 Japanese female subjects who had a typical Japanese complexion, with a Fitzpatrick skin typing score of III (which is the Japanese average), ages of more than 40 years, a life style with little sun exposure, and at least one SL on both sides of their faces with a similar pigmentation level were recruited and enrolled in this study. The subjects were instructed to apply lotions with or without 28% AGAC (test lotion and placebo lotion, respectively) twice a day for 24 weeks on the entire right and left side of their face. They were also instructed in a double-blind manner to apply the test lotion on one side of their face and the placebo lotion on the opposite side. 

### 5.3. Evaluation of Pigmentation Level

Evaluations and measurements of pigmentation levels were performed on a previously selected SL and one NLS area on each half of the face of each subject. Because of the limited number of volunteers with two or more SLs with a similar pigmentation level on each half of their face and due to the paired comparison test between the right and left halves of the faces of each subject, only one SL (over 20.0 mm^2^) with a similar pigmentation level and one NLS area on the right and left sides of the face of each subject were selected as evaluation sites [47]. The pigmentation levels of a previously selected SL and one NLS area were evaluated using a photo-scale ranging from 1.0 to 5.0 by a trained dermatologist (KN). The pigmentation levels in the selected SL and a NLS area were also measured using a color difference meter CM-700 d with a probe area of 7 mm^2^ (Konica Minolta Japan, Inc, Tokyo, Japan) to determine the L values [47] and using a Mexameter MX18 with a probe area of 19.6 mm^2^ (Courage + Khazaka Electronic GmbH, Cologne, Germany) [51,52,53,54] to measure the melanin index (MI) values at 0, 12 and 24 weeks of treatment. The average values of 5 measurements at each SL and NLS area on each side of the face of each subject were used as data for this study.

### 5.4. General Clinical Evaluation

A general clinical evaluation was performed by a trained dermatologist (KN) on the entire faces of the 27 subjects with SLs to check for dryness, scaling, erythema, papules, stinging sensations or itchiness, with scores of 1 to 5 corresponding to none, weak, mild, moderate and severe, at 0, 12 and 24 weeks of treatment. 

### 5.5. Evaluation of Skin Redness Level

For the evaluation of skin irritation, skin redness levels in a selected SL and one NLS area (the same SL and NLS area as evaluated for pigmentation levels) were evaluated using a Mexameter MX18 as erythema index values, which reflect skin redness levels based on hemoglobin levels related to skin blood flow [51]. The average values of 5 measurements at each SL and NLS area on the face of each subject were used as data for this study.

### 5.6. Statistics

All data are expressed as means ± standard deviation (SD) unless noted otherwise. GraphPad Prism 10 from Graph Pad Software (Boston, MA, USA) was used as the software for statistical analysis. For pairwise comparisons, a *t* test was used. ANOVA and Tukey’s comparison test were used to evaluate pigmentation scores and changes in L values, MI values and erythema index values. Friedman’s test and Dunn’s multiple comparisons test were used for skin scoring. *p* values < 0.05 are considered statistically significant.

## Figures and Tables

**Table 1:** Full ingredient list of the test lotion (containing 28% AGAC) and the placebo lotion (without AGAC).

Ingredients
With or without 28% Ascorbyl Glucoside–Arginine Complex (AGAC), Water, Propanediol, Butylene Glycol (BG), Betaine, Pentylene Glycol, 1,2-Hexanediol, PEG-60 Hydrogenated Castor Oil, PEG-9M, Citric Acid, Sodium Citrate, Glycerin, Sphingolipids, Hydrolyzed Elastin, Hydrolyzed Collagen

## Data Availability

All relevant data are included in the manuscript.

## Abbreviations

SLs, solar lentigos; AGAC, ascorbyl glucoside–arginine complex; EDN, endothelin; SCF, stem cell factor; NLS, non-lesional surrounding skin; AG, ascorbyl glucoside; AMP, L-ascorbate-2-phosphate Mg; APS, L-ascorbate-2-phosphate trisodium salt; HQ, hydroquinone; L, lightness; Δ L, increased L value; MI, melanin index; Δ MI, decreased MI value; TNF, tumor necrosis factor; UVB, ultraviolet B.

## References

[1] Imokawa G. (2019). Melanocyte Activation Mechanisms and Rational Therapeutic Treatments of Solar Lentigos. Int. J. Mol. Sci..

[2] Rajaratnam R., Halpern J., Salim A., Emmett C. (2010). Interventions for melasma. Cochrane Database Syst. Rev..

[3] Arndt K.A., Fitzpatrick T.B. (1965). Topical use of hydroquinone as a depigmenting agent. JAMA.

[4] Fitzpatrick T.B., Arndt K.A., El Mofty A.M., Pathak M.A. (1966). Hydroquinone and psoralens in the therapy of hypermelanosis and vitiligo. Arch. Dermatol..

[5] Kligman A.M., Willis I. (1975). A new formula for depigmenting human skin. Arch. Dermatol..

[6] Heilgemeir G.P., Balda B.R. (1981). Irreversible toxic depigmentation. Observations following use of hydroquinonemonobenzylether-containing skin bleaching preparations. MMW Munch. Med. Wochenschr..

[7] Halder R.M., Richards G.M. (2004). Topical agents used in the management of hyperpigmentation. Skin Ther. Lett..

[8] Cheong K.A., Kim H.J., Kim J.Y., Kim C.H., Lim W.S., Noh M., Lee A.Y. (2014). Retinoic acid and hydroquinone induce inverse expression patterns on cornified envelope-associated proteins: Implication in skin irritation. J. Dermatol. Sci..

[9] Zheng Y., Du X., Zhang L., Jia T., Zhang H., Peng B., Hao Y., Tong Z., Che D., Geng S. (2023). Hydroquinone-induced skin irritant reaction could be achieved by activating mast cells via mas-related G protein-coupled receptor X2. Exp. Dermatol..

[10] Kim G.H., Cheong K.A., Lee A.Y. (2017). Increased Skin Irritation by Hydroquinone and Rsetinoic Acid Used in Combination. Ann. Dermatol..

[11] McKesey J., Tovar-Garza A., Pandya A.G. (2020). Melasma Treatment: An Evidence-Based Review. Am. J. Clin. Dermatol..

[12] Tai Y., Wang C., Wang Z., Liang Y., Du J., He D., Fan X., Jordt S.E., Liu B. (2017). Involvement of Transient Receptor Potential Cation Channel Member A1 activation in the irritation and pain response elicited by skin-lightening reagent hydroquinone. Sci. Rep..

[13] Searle T., Al-Niaimi F., Ali F.R. (2021). Hydroquinone: Myths and reality. Clin. Exp. Dermatol..

[14] Cabanes J., Chazarra S., Garcia-Carmona F. (1994). Kojic acid, a cosmetic skin whitening agent, is a slow-binding inhibitor of catecholase activity of tyrosinase. J. Pharm. Pharmacol..

[15] Kumar K.J., Vani M.G., Wang S.Y., Liao J.W., Hsu L.S., Yang H.L., Hseu Y.C. (2013). In vitro and in vivo studies disclosed the depigmenting effects of gallic acid: A novel skin lightening agent for hyperpigmentary skin diseases. Biofactors.

[16] Gonçalez M.L., Corrêa M.A., Chorilli M. (2013). Skin delivery of kojic acid-loaded nanotechnology-based drug delivery systems for the treatment of skin aging. Biomed. Res. Int..

[17] Ki D.H., Jung H.C., Noh Y.W., Thanigaimalai P., Kim B.H., Shin S.C., Jung S.H., Cho C.W. (2013). Preformulation and formulation of newly synthesized QNT3-18 for development of a skin whitening agent. Drug Dev. Ind. Pharm..

[18] Breathnach A.C., Nazzaro-Porro M., Passi S., Zina G. (1989). Azelaic acid therapy in disorders of pigmentation. Clin. Dermatol..

[19] Verallo-Rowell V.M., Verallo V., Graupe K., Lopez-Villafuerte L., Garcia-Lopez M. (1989). Double-blind comparison of azelaic acid and hydroquinone in the treatment of melasma. Acta Derm. Venereol. Suppl. (Stock.).

[20] Huang C.H., Sung H.C., Hsiao C.Y., Hu S., Ko Y.S. (2013). Transdermal delivery of three vitamin C derivatives by Er:YAG and carbon dioxide laser pretreatment. Lasers Med. Sci..

[21] Won Y.K., Loy C.J., Randhawa M., Southall M.D. (2014). Clinical efficacy and safety of 4-hexyl-1,3-phenylenediol for improving skin hyperpigmentation. Arch. Dermatol. Res..

[22] Son K.H., Heo M.Y. (2013). The evaluation of depigmenting efficacy in the skin for the development of new whitening agents in Korea. Int. J. Cosmet. Sci..

[23] Chen Y.S., Lee S.M., Lin C.C., Liu C.Y., Wu M.C., Shi W.L. (2013). Kinetic study on the tyrosinase and melanin formation inhibitory activities of carthamus yellow isolated from Carthamus tinctorius L.. J. Biosci. Bioeng..

[24] Hsieh P.W., Chen W.Y., Aljuffali I.A., Chen C.C., Fang J.Y. (2013). Co-drug strategy for promoting skin targeting and minimizing the transdermal diffusion of hydroquinone and tranexamic acid. Curr. Med. Chem..

[25] Tse T.W., Hui E. (2013). Tranexamic acid: An important adjuvant in the treatment of melasma. J. Cosmet. Dermatol..

[26] Eimpunth S., Wanitphadeedecha R., Manuskiatti W. (2013). A focused review on acne-induced and aesthetic procedure-related postinflammatory hyperpigmentation in Asians. J. Eur. Acad. Dermatol. Venereol..

[27] Pillaiyar T., Manickam M., Namasivayam V. (2017). Skin whitening agents: Medicinal chemistry perspective of tyrosinase inhibitors. J. Enzym. Inhib. Med. Chem..

[28] Saeedi M., Eslamifar M., Khezri K. (2019). Kojic acid applications in cosmetic and pharmaceutical preparations. Biomed. Pharmacother..

[29] Desai S., Ayres E., Bak H., Manco M., Lynch S., Raab S., Du A., Green D., Skobowiat C., Wangari-Talbot J. (2019). Effect of a Tranexamic Acid, Kojic Acid, and Niacinamide Containing Serum on Facial Dyschromia: A Clinical Evaluation. J. Drugs Dermatol..

[30] Thawabteh A.M., Jibreen A., Karaman D., Thawabteh A., Karaman R. (2023). Skin Pigmentation Types, Causes and Treatment—A Review. Molecules.

[31] Mota S., Rosa G.P., Barreto M.C., Garrido J., Sousa E., Cruz M.T., Almeida I.F., Quintas C. (2023). Comparative Studies on the Photoreactivity, Efficacy, and Safety of Depigmenting Agents. Pharmaceuticals.

[32] Matsunaga K., Suzuki K., Ito A., Tanemura A., Abe Y., Suzuki T., Yoshikawa M., Sumikawa Y., Yagami A., Masui Y. (2021). Rhododendrol-induced leukoderma update I: Clinical findings and treatment. J. Dermatol..

[33] Inoue S., Katayama I., Suzuki T., Tanemura A., Ito S., Abe Y., Sumikawa Y., Yoshikawa M., Suzuki K., Yagami A. (2021). Rhododendrol-induced leukoderma update II: Pathophysiology, mechanisms, risk evaluation, and possible mechanism-based treatments in comparison with vitiligo. J. Dermatol..

[34] Madhogaria S., Ahmed I. (2010). Leucoderma after use of a skin-lightening cream containing kojic dipalmitate, liquorice root extract and Mitracarpus scaber extract. Clin. Exp. Dermatol..

[35] Sasaki M., Kondo M., Sato K., Umeda M., Kawabata K., Takahashi Y., Suzuki T., Matsunaga K., Inoue S. (2014). Rhododendrol, a depigmentation-inducing phenolic compound, exerts melanocyte cytotoxicity via a tyrosinase-dependent mechanism. Pigment. Cell Melanoma Res..

[36] Elmore A.R. (2005). Final report of the safety assessment of L-Ascorbic Acid, Calcium Ascorbate, Magnesium Ascorbate, Magnesium Ascorbyl Phosphate, Sodium Ascorbate, and Sodium Ascorbyl Phosphate as used in cosmetics. Int. J. Toxicol..

[37] Kameyama K., Sakai C., Kondoh S., Yonemoto K., Nishiyama S., Tagawa M., Murata T., Ohnuma T., Quigley J., Dorsky A. (1996). Inhibitory effect of magnesium L-ascorbyl-2-phosphate (VC-PMG) on melanogenesis in vitro and in vivo. J. Am. Acad. Dermatol..

[38] Kobayashi S., Takehana M., Itoh S., Ogata E. (1996). Protective effect of magnesium-L-ascorbyl-2 phosphate against skin damage induced by UVB irradiation. Photochem. Photobiol.

[39] Murtaza F., Bangash A.R., Khushdil A., Noor S.M. (2016). Efficacy of Trichloro-Acetic Acid Peel Alone Versus Combined Topical Magnesium Ascorbyl Phosphate for Epidermal Melasma. J. Coll. Physicians Surg. Pak..

[40] Shaikh Z.I., Mashood A.A. (2014). Treatment of refractory melasma with combination of topical 5% magnesium ascorbyl phosphate and fluorescent pulsed light in Asian patients. Int. J. Dermatol..

[41] Silva G.M., Maia Campos P.M. (2000). Histopathological, morphometric and stereological studies of ascorbic acid and magnesium ascorbyl phosphate in a skin care formulation. Int. J. Cosmet. Sci..

[42] Wang P.C., Huang Y.L., Hou S.S., Chou C.H., Tsai J.C. (2013). Lauroyl/palmitoyl glycol chitosan gels enhance skin delivery of magnesium ascorbyl phosphate. J. Cosmet. Sci..

[43] Yamamoto K., Shichiri H., Ishida T., Kaku K., Nishioka T., Kume M., Makimoto H., Nakagawa T., Hirano T., Bito T. (2017). Effects of Ascorbyl-2-phosphate Magnesium on Human Keratinocyte Toxicity and Pathological Changes by Sorafenib. Biol. Pharm. Bull..

[44] Foco A., Gasperlin M., Kristl J. (2005). Investigation of liposomes as carriers of sodium ascorbyl phosphate for cutaneous photoprotection. Int. J. Pharm..

[45] Nayama S., Takehana M., Kanke M., Itoh S., Ogata E., Kobayashi S. (1999). Protective effects of sodium-L-ascorbyl-2 phosphate on the development of UVB-induced damage in cultured mouse skin. Biol. Pharm. Bull..

[46] Khan H., Akhtar N., Ali A., Khan H.M.S., Sohail M., Naeem M., Nawaz Z. (2016). Physical and Chemical Stability Analysis of Cosmetic Multiple Emulsions Loaded with Ascorbyl Palmitate and Sodium Ascorbyl Phosphate Salts. Acta Pol. Pharm..

[47] Ishikawa Y., Niwano T., Hirano S., Numano K., Takasima K., Imokawa G. (2019). Whitening effect of L-ascorbate-2-phosphate trisodium salt on solar lentigos. Arch. Dermatol. Res..

[48] Johnson W., Bergfeld W.F., Belsito D.V., Klaassen C.D., Liebler D.C., Marks J.G., Peterson L.A., Shank R.C., Slaga T.J., Snyder P.W. (2024). Safety Assessment of Ascorbyl Glucoside and Sodium Ascorbyl Glucoside as Used in Cosmetics. Int. J. Toxicol..

[49] Jesus A., Correia-da-Silva M., Confraria C., Silva S., Brites G., Sebastião A.I., Carrascal M., Pinto M., Cidade H., Costa P. (2024). Persulfated Ascorbic Acid Glycoside as a Safe and Stable Derivative of Ascorbic Acid for Skin Care Application. Molecules.

[50] Nakano M., Murakami A., Yamamoto A., Kubo N.T. (2023). The safety assessment of stable type neural vitamin C derivative (MIRAVC). Shinryo Shinyaku (Clin. Pract. New Drugs).

[51] Feather J.W., Ellis D.J., Leslie G. (1988). A portable reflectometer for the rapid quantification of cutaneous haemoglobin and melanin. Phys. Med. Biol..

[52] Dawson J.B., Barker D.J., Ellis D.J., Grassam E., Cotterill J.A., Fisher G.W., Feather J.W. (1980). A theoretical and experimental study of light absorption and scattering by in vivo skin. Phys. Med. Biol..

[53] Feather J.W., Hajizadeh-Saffar M., Leslie G., Dawson J.B. (1989). A portable scanning reflectance spectrophotometer using visible wavelengths for the rapid measurement of skin pigments. Phys. Med. Biol..

[54] Kollias N., Baqer A. (1985). Spectroscopic characteristics of human melanin in vivo. J. Investig. Dermatol..

[55] Ohshima H., Takiwaki H. (2008). Evaluation of dark circles of the lower eyelid: Comparison between reflectance meters and image processing and involvement of dermal thickness in appearance. Skin Res. Technol..

[56] Imokawa G. (1990). Analysis of carbohydrate properties essential for melanogenesis in tyrosinases of cultured malignant melanoma cells by differential carbohydrate processing inhibition. J. Investig. Dermatol..

